# Using food composition tables to estimate decreases in sodium intake due to the reformulation of packaged and ultra-processed foods in a young population in South Africa

**DOI:** 10.1017/S1368980024002519

**Published:** 2024-12-26

**Authors:** Alexandra Ross, Elizabeth C Swart, Joelaine Chetty, Malory Jumat, Tamryn Frank, Averalda van Graan

**Affiliations:** 1 Department of Nutrition, Gillings School of Global Public Health and Carolina Population Center, University of North Carolina, Chapel Hill 27599-7400, NC, USA; 2 Department of Dietetics and Nutrition, University of the Western Cape, Cape Town, Republic of South Africa; 3 DST/NRF Center of Excellence in Food Security, University of the Western Cape, Cape Town, Republic of South Africa; 4 South African Medical Research Council, Cape Town, Republic of South Africa

**Keywords:** Sodium, Hypertension, Regulation, Policy, Food composition tables

## Abstract

**Objective:**

In response to increasing hypertension rates, South Africa implemented a regulation which set a maximum total Na content for certain packaged food categories. We assess changes in reported Na intake among 18–39 years old adults living in one township in the Western Cape as a result of the implementation of the regulation in 2016.

**Design:**

By linking one set of 24-h dietary recall data to two versions of the South Africa Food Composition Database which reflect the pre-regulation and post-regulation periods, we calculated changes in Na intake due to reformulation of food products, not behaviour change. We statistically tested differences in mean consumption in this sample with paired *t* tests.

**Setting:**

Langa, Western Cape, South Africa

**Participants:**

Surveyed participants were residents of Langa between 18 and 39 years old (*n* 2148)

**Results:**

Before and after the implementation of the regulation, there was a statistically significant decrease in the estimated Na intake among adults of 189·4 mg (137·5, 241·4; *P* = 0·00). Reported Na from cured meat (such as Russians) and certain types of soup powder, cereals and salted peanuts had a 9 to 33 per cent lower calculated Na consumption.

**Conclusions:**

Our conclusions show that independent of any behavioural changes on the part of consumers, it is possible to lower Na intake by using regulations to induce food manufacturers to lower the Na levels in their products. As countries explore similar regulatory strategies, this work can add to that body of evidence to inform policies to improve the food system.

Excessive consumption of Na can lead to the development of hypertension and subsequent risk for CVD, stroke and other metabolic conditions^(1–4)^. South Africa has high hypertension rates, and trends over time revealed this prevalence is increasing, with higher risk among males, those that identify as black, live in an urban area and those with self-reported high blood cholesterol^(5,6)^. These increasing hypertension rates in South Africa have resulted in increased stroke and CVD, adding a major strain to the healthcare system and to the government health budget^(7)^.

As most dietary Na in South Africa comes from packaged and prepared foods such as breads, dried and processed meats, and ultra-processed snack foods, the reduction of Na in these products can be an effective strategy to reduce overall consumption of Na and risk of CVD. Thus, in response to the increasing concern over consumption of these foods, in 2013 the South African National Department of Health passed new mandatory regulation limits on the quantity of Na used in selected processed foods to be implemented starting in 2016^(8,9)^. This regulation, called the *Reduction of Sodium in Certain Foodstuffs and Related Matters*, set a maximum total Na content for certain packaged food products, for thirteen food categories (Table 1). The Na reduction goals were targeted across several industrially produced food categories that utilised high Na levels in their products. Mandatory guidelines for Na levels limited specific foods with high consumption rates in South Africa, including bread, margarine and soup mixes. It had a phased approach whereby the food manufacturers were instructed to reduce Na levels in two waves by end of June 2016 and end of June 2019. For certain processed meat products, the limits were revised and the implementation date were amended to 30 April 2020.

**Table 1.** Food groups and sodium targets under regulations relating to reduction of sodium in certain foodstuffs and related matters of 2013 (R.214) and amendments (no.989 of 2016; no.1071 of 2017; no.812 of 2019) in South Africa

Food category	Max total Na per 100 g as sold	Max total Na per 100 g as sold
	Phase 1 Target: 2016	Phase 2 Target: 2019
Bread	400 mg Na	380 mg Na
All breakfast cereals and porridges, whether ready-to-eat, instant or cook up, hot or cold	500 mg Na	400 mg Na
All fat spreads and butter spreads	550 mg Na	450 mg Na
Ready-to-eat savoury snacks, excluding salt- and vinegar-flavoured savoury snacks	800 mg Na	700 mg Na
Flavoured potato crisps, excluding salt- and vinegar-flavoured potato crisps	650 mg Na	550 mg Na
Flavoured, ready-to-eat, savoury snacks and potato crisps – salted and salt-and-vinegar only	1000 mg Na	850 mg Na
Processed meats – cured	1300 mg Na (30 Mar 2017)	1150 mg Na (30 Apr 2020)
Processed meats – uncured	850 mg Na	650 mg Na 30 Apr 2020
Raw-processed meat sausages (all types) and similar products	800 mg Na	600 mg Na 30 Apr 2020
Dry savoury powders (not the instant type)	5500 mg Na	3500 mg Na
Dry gravy powders and savoury sauce powders	3500 mg Na	2000 mg Na
Dry savoury powders with dry instant noodles to be mixed with a liquid	1500 mg Na	800 mg Na
Stock cubes, stock powders, stock granules, stock emulsions, stock pastes or stock jellies	18 000 mg Na	15 000 mg Na

By implementing this Na reduction, South Africa is one of the first countries in the world (followed by Argentina in December 2013) to regulate Na consumption at the manufacturing level for several commonly consumed industrially produced foods^(10)^. The food environment within South Africa is rapidly changing, with cheaper, energy-dense, ultra-processed and unhealthy food options becoming the food of choice for many^(11–14)^. A recent study by Charlton *et al.* reported that the population Na intake of South Africans reduced by 1·16 g salt per d between 2015 and 2018/early 2019, with the median salt intake in their sample group being 6·1 g of salt per d after the first phase of the Na reduction strategy^(15)^. The same study found that younger people had substantially higher salt intakes than older adults, with a median salt intake of those aged 18–49 years estimated to be 7·8 g/d^(15)^. However, no studies have shown how the reformulation of these food products changed Na consumption, without the influence of behaviour change due to increased awareness of the regulation or rising CVD rates. Furthermore, studies should consider the potential for these reformulations among younger, low-income, high packaged and processed food consumers, as the regulation may have a higher impact on the Na consumption and subsequent health implications in these populations.

To bridge the existing research gaps regarding the effects of Na regulations on low-income subpopulations, the objective of this study is to evaluate the changes in Na intake among young adults aged 18–39 years residing in a low-income township in the Western Cape, both before and after the Na regulation was implemented in 2016. By applying food composition tables (FCT) reflecting formulation of packaged and ultra-processed foods, we estimated how the Na regulation resulted in reformulation of foods and thus changes in Na intake, not accounting for changes in knowledge and subsequent nutrition-related behaviours. Given the anticipated reformulation of products containing an excessive amount of Na that are known to be highly consumed in this population, we hypothesised that there will be a significant reduction in the consumption of Na in this population due to the reformulation of packaged foods.

## Methods

### Updating sodium content in food composition tables

Previous work has found that an estimated two-thirds of packaged foods in the South African Food Composition Database were reformulated to comply with the maximums set in the first phase of the regulation^(16–18)^. To update the FCT for the country and assess the Na contents of foods, The South African Food Data System (SAFOODS) division of the South African Medical Research Council (SAMRC) directly sourced nutrient data via food company representatives. Locally accredited food laboratories chemically analysed data were used for updating the FCT with newer Na values and other components. Standardised food compilation data quality assurance methods were applied to these data prior to updating the values in the FCT used in this analysis: the 2017 FCT which represents the Na content of foods pre-regulation which were finalised prior to the 2016 regulation implementation date, and the 2021 version which represents the Na content of foods post-regulation^(19,20)^. Data from nutrient facts panels or labels were not used to update the nutrient information in either version of the FCT.

### 24-h recall collection

We analysed single-day 24-h dietary recalls from a cross-sectional survey of young township adults aged 18–39 years living in the lower income Langa township near Cape Town, South Africa. Data for this analysis were collected in February–March 2018 (*n* 2148). All participants gave written, informed consent to participate in the dietary recalls, which were collected as part of an evaluation of another policy change. Systematic door-to-door sampling was conducted and one randomly selected consenting adult between the ages of 18–39 years per household was included in the study. The interviewer-administered household questionnaire was completed digitally using android phones and included a geolocation. For the diet assessment, 24-h diet recalls were conducted by interviewers with nutrition training. Individual participants reported what foods and drinks were eaten, how foods and beverages were prepared, whether anything was added and the quantity consumed. Data collectors did not prompt about addition of salt in meals at the table or in the cooking process. A multiple-pass approach, including detailed prompting, was used to enhance completeness. Only one diet assessment was completed for one individual within a given household.

Dietary recalls were coded by trained data capturers with high levels of previous nutrition experience. The SAMRC Food Quantities Manual and Composition Tables were used for coding. An extensive assumptions manual was developed to ensure coding decisions were made in a standardised manner.

### Analytical approach

All analyses were conducted in Stata, version 16. Our key outcome variable was change in mg of Na for all foods reported in the 24-h recalls. By linking 2018 dietary intake data only to FCT created to reflect the pre-regulation period and then separately to the post-regulation period, we are able to calculate changes in Na intake due to reformulation, not behaviour change (since we are keeping the diet data consistent). Specifically, Na content of foods reported were compared with Na contents using the two versions of the South African Food Composition Database. Differences in nutrient values were statistically tested with paired *t* tests to compare the mean Na values of this sample between the two time points. Subgroup analyses were performed to look at changes in Na consumption specifically among participants that were identified as ‘high’ consumers of Na in the pre-regulation period. High consumers were defined as those that consume more than the WHO recommended 2000 mg of Na per d^(21)^.

## Results

### Changes in reported intake

Using the 2021 FCT values (representing Na values post-regulation), the mean estimated Na intake was 1780 mg per d for men and 1515 mg per d for women. This is a decrease from the 1866 mg/d for men and 1620 mg/d for women when applying the pre-regulation FCT values (representing Na values pre-regulation). The mean estimated Na intake measured using pre-regulation and post-regulation values was under the recommended maximum intake of 2000 mg per d set by the WHO and the South African government for both men and women (Table 2). Between pre-regulation and post-regulation, there was a statistically significant decrease in the estimated Na intake among adults of 93·6 mg (76·5, 110·7; *P* = 0·00).

**Table 2.** Mean change in sodium intake values (mg) for men and women in one township in Western Cape, South Africa

	Men	Women	Total	Difference	
All participants (*n* 2162)					
Pre-regulation	1866·3	1619·9	1703·8	93·6	76·5, 110·7^*^
Post-regulation	1779·7	1515·0	1610·1		
High consumers (>WHO recommended 2000 mg/d) (*n* 651)					
Pre-regulation	3373·8	3259·6	3305·8	189·4	137·5, 241·4^*^
Post-regulation	3187·8	2989·4	3116·4		

* Indicates statistical significance (*P* < 0·05).

Thirty per cent of respondents reported consuming more than the recommended 2000 mg before the implementation of the regulation. Among this population of high Na consumers, the mean estimated Na intake was 3188 mg per d for men and 2989 mg per d for women, as opposed to pre-regulation values of 3374 mg/d for men and 3260 mg/d for women. Between pre-regulation and post-regulation, there was a statistically significant decrease in the estimated Na intake among high Na consumers of 189·4 mg (137·5, 241·4; *P* = 0·00).

### Changes by food category

In our study population, the food sub-categories that contributed the most amount of Na in the diet were Russians, instant noodles, soup from powder mixes, grilled sausages and brown bread (Table 3).

**Table 3.** Difference in sodium consumption (mg) by applying pre- and post-regulation food composition table sodium values to same dietary intake data

Food category	Average grams reported as consumed	Average Na consumed per Meal using pre-regulation Na values (mg)	Average Na consumed per meal using post-regulation Na values (mg)	% difference
Cereals and cereal products	201·5	358·68	302·01	−16 %
Breakfast cereal – corn flakes, plain	104·3	1355·95	515·47	−62 %
Breakfast cereal – Rice Crispies	178·6	2362·50	580·36	−75 %
Bread/rolls, brown (fortified)	130·17	856·18	843·50	−1 %
Bread/rolls, white (fortified)	126·85	824·02	828·37	1 %
Vegetables	63·7	33·97	33·92	0 %
Fruits	152·7	5·03	5·01	0 %
Legumes and legume products	91·2	170·47	158·97	−7 %
Nuts and seeds	43·7	105·41	70·53	−33 %
Salted peanuts	71·0	307·43	227·2	−29 %
Milk and milk products	158·2	127·68	131·84	3 %
Eggs	116·7	142·05	139·78	−2 %
Meat and meat products	67·1	219·10	212·23	−3 %
Salami, beef/pork, including Russians	91·8	1769·36	1612·42	−9 %
Sausage, beef, grilled	154·1	1858·47	1848·96	−1 %
Sausage, beef, dry, including droëwors	130·9	2114·74	2114·74	0 %
Sausage, beef and pork, grilled, including boerewors	143·1	1322·10	1329·27	1 %
Fish and seafood	120·6	349·66	356·10	2 %
Fats and oils	20·5	126·61	118·85	−6 %
Sugar, syrups and sweets	180·8	14·24	15·89	12 %
Soups, sauces, seasonings and flavourings	57·1	418·78	377·24	−10 %
Soup, powder, onion	41·4	3708·20	3324·83	−10 %

In comparing the values from the pre- and post-regulation FCT, the Na content from salami meat and in certain types of soup powder was on average 9 to 10 per cent lower, and nuts and seeds had a 10 to 33 per cent lower Na content (given that they were classified as a savoury snack under the regulation). We found that cereal products overall had a 16 % reduction in Na values driven by vast reduction in certain branded products. As an example, Rice Crispies in the pre-regulation FCT reported 1323 mg per 100 g, whilst newer reference values listed in the 2021 FCT reported 330 mg per 100 g, which is approximately four times more Na in earlier years reported by the manufacturer. Na content in other categories of foods had not changed significantly.

## Discussion

When we applied Na values from FCT reflecting the pre- and post-Na regulation period to dietary intake data from a low-income adult population, we observed a statistically significant difference in estimated Na intakes due to reformulation of packaged and ultra-processed foods. This study was done as a theoretical assessment to postulate effects of the regulation on Na intake, so there are limitations in interpretation. Awareness of the Na regulation could change food choice and consumption behaviours, which could be assessed with diet recalls performed before and after the implementation of the regulation on the same individuals. Our conclusions show that independent of any behavioural changes on the part of consumers, it is possible to lower Na intake by using regulations to induce food manufacturers to lower the Na levels in their products. We also showed that a policy such as this regulation may have a greater impact for high Na consumers.

A modelling study that informed the design of the Na legislation in South Africa estimated that reducing current daily Na intake by 850 mg per person, per day was a clinically relevant amount to show a decrease in cardiovascular mortality on a population level^(22)^. Although we observed a decrease between 93·6 and 189 mg of Na, the values we saw represent an *underestimation* of the true Na intake in this population because addition of table salt was not prompted during the collection of the 24-h dietary recalls. For the 2017 FCT representing pre-implementation values, most values were updated after 2013 when the regulation was announced. The comparisons may underestimate the true level of change as ‘early adopted’ companies may have reformulated shortly after the publishing of the draft regulations. Many FCT codes represent composite values of many foods – including some that are a mix of regulated (e.g. grilled sausage) and unregulated products (boerewors) which limits our ability to observe accurate changes in Na. Additionally, updates to the Na contents of the 2021 FCT excludes recipes and foods prepared outside the home, which include highly consumed items such as chips (French fries). Furthermore, because differences in behaviour were not assessed, we cannot tell if participants in the post-regulation period might have made up for the loss of Na in packaged foods by adding salt at the table. However, the differences in Na intake observed specifically from the consumption of packaged and ultra-processed food intake (93·6 overall and 189·4 in high consumers) will likely be similar if these behaviour changes were introduced.

### Conclusion

Subsequent to South Africa becoming the first sub-Saharan African country to implement such a Na regulation, other countries around the world are exploring similar strategies to reduce the growing rates of hypertension and CVD. Our findings suggest that regulatory approaches to induce manufacturers to reduce Na in their products can be a useful tool for improving population health. This work can add to that body of evidence, to inform policies to improve this imbalance in the food system in similar contexts.

## References

[1] Meneton P , Jeunemaitre X , de Wardener HE et al. (2005) Links between dietary salt intake, renal salt handling, blood pressure, and cardiovascular diseases. Physiol Rev 85, 679–715. 15788708 10.1152/physrev.00056.2003

[2] Yusuf S , Joseph P , Rangarajan S et al. (2020) Modifiable risk factors, cardiovascular disease, and mortality in 155 722 individuals from 21 high-income, middle-income, and low-income countries (PURE): a prospective cohort study. Lancet 395, 795–808. 31492503 10.1016/S0140-6736(19)32008-2 PMC8006904

[3] He FJ & MacGregor GA (2010) Reducing population salt intake worldwide: from evidence to implementation. Prog Cardiovasc Dis 52, 363–382. 20226955 10.1016/j.pcad.2009.12.006

[4] Buttar HS , Li T & Ravi N (2005) Prevention of cardiovascular diseases: role of exercise, dietary interventions, obesity and smoking cessation. Exp Clin Cardiol 10, 229. 19641674 PMC2716237

[5] Mungal-Singh V (2012) Lifestyle changes for hypertension. S Afr Family Pract 54, S12–S16. doi: 10.1080/20786204.2012.10874203.

[6] Kandala N-B , Nnanatu CC , Dukhi N et al. (2021) Mapping the burden of hypertension in South Africa: a comparative analysis of the National 2012 SANHANES and the 2016 Demographic and Health Survey. Int J Environ Res Public Health 18, 5445. 34069668 10.3390/ijerph18105445 PMC8160950

[7] Pestana J , Steyn K , Leiman A et al. (1996) The direct and indirect costs of cardiovascular disease in South Africa in 1991. S Afr Med J 86, 679–684. 8764427

[8] Department of Health (2013) Reduction of Sodium in Certain Foodstuffs and Related Matters of 2013 (R.214). https://www.gov.za/sites/default/files/gcis_document/201710/41164gon1071.pdf (accessed July 2022).

[9] Webster J , Santos JA , Hogendorf M et al. (2022) Implementing effective salt reduction programs and policies in low-and middle-income countries: learning from retrospective policy analysis in Argentina, Mongolia, South Africa and Vietnam. Public Health Nutr 25, 805–816. 34384514 10.1017/S136898002100344X PMC9991649

[10] Boletin Oficial de la Republica Argentina Ley 26.905 (2013) Promoción de la Reducción del Consumo de Sodio en la Población (Promotion of Sodium Reduction in the Population). https://www.boletinoficial.gob.ar/detalleAviso/primera/99389/20131216?busqueda=1 (accessed 07 July 2022).

[11] Temple NJ & Steyn NP (2011) The cost of a healthy diet: a South African perspective. Nutrition 27, 505–508. 21074973 10.1016/j.nut.2010.09.005

[12] Nel JH & Casey A (2003) Secondary data analyses of dietary surveys undertaken in South Africa to determine usual food consumption of the population. Public Health Nutr 6, 631–644. 14552664 10.1079/phn2003482

[13] Oldewage-Theron WH & Slabbert TJ (2008) Impact of food and nutrition interventions on poverty in an informal settlement in the Vaal Region of South Africa. Proc Nutr Soc 67, 91–97. 18234136 10.1017/S002966510800606X

[14] Armstrong ME , Lambert MI & Lambert EV (2011) Secular trends in the prevalence of stunting, overweight and obesity among South African children (1994–2004). Eur J Clin Nutr 65, 835–840. 21505505 10.1038/ejcn.2011.46

[15] Charlton KE , Corso B , Ware L et al. (2021) Effect of South Africa’s interim mandatory salt reduction programme on urinary sodium excretion and blood pressure. Prev Med Rep 23, 101469. 34381665 10.1016/j.pmedr.2021.101469 PMC8333157

[16] Peters SA , Dunford E , Ware LJ et al. (2017) The sodium content of processed foods in South Africa during the introduction of mandatory sodium limits. Nutrients 9, 404. 28425938 10.3390/nu9040404 PMC5409743

[17] Swanepoel B , Malan L , Myburgh PH et al. (2017) Sodium content of foodstuffs included in the sodium reduction regulation of South Africa. J Food Compos Anal 63, 73–78.

[18] van der Westhuizen B , Frank T , Karim SA et al. (2023) Determining food industry compliance to mandatory sodium limits: successes and challenges from the South African experience. Public Health Nutr 1–8, 26(11), 2551–2558. 37070406 10.1017/S1368980023000757 PMC10641639

[19] SAFOODS (2018) *SAMRC Food Quantities Manual for South Africa*, 3rd ed. (ebook). Cape Town: South African Medical Research Council. http://safoods.mrc.ac.za (accessed July 2022).

[20] SAFOODS (2022) *SAMRC Food Composition Tables for South Africa*, 5th ed. (ebook). Cape Town: South African Medical Research Council. http://safoods.mrc.ac.za (accessed July 2022).

[21] Organization WH (2007) Reducing salt intake in populations: report of a WHO forum and technical meeting, 5–7 October 2006, Paris, France.

[22] Bertram MY , Tollman S , Hofman KJ et al. (2012) Reducing the sodium content of high-salt foods: effect on cardiovascular disease in South Africa. S Afr Med J 102, 743–745. 22958695 10.7196/samj.5832

